# Neural signals implicated in the processing of appetitive and aversive events in social and non-social contexts

**DOI:** 10.3389/fnsys.2022.926388

**Published:** 2022-08-03

**Authors:** Daniela Vázquez, Kevin N. Schneider, Matthew R. Roesch

**Affiliations:** ^1^Department of Psychology, University of Maryland, College Park, College Park, MD, United States; ^2^Neuroscience and Cognitive Science Program, University of Maryland, College Park, College Park, MD, United States

**Keywords:** anterior cingulate cortex, dopamine, social behavior, salience, attention, reward, aversive, electrophysiology

## Abstract

In 2014, we participated in a special issue of Frontiers examining the neural processing of appetitive and aversive events. Specifically, we reviewed brain areas that contribute to the encoding of prediction errors and value versus salience, attention and motivation. Further, we described how we disambiguated these cognitive processes and their neural substrates by using paradigms that incorporate both appetitive and aversive stimuli. We described a circuit in which the orbitofrontal cortex (OFC) signals expected value and the basolateral amygdala (BLA) encodes the salience and valence of both appetitive and aversive events. This information is integrated by the nucleus accumbens (NAc) and dopaminergic (DA) signaling in order to generate prediction and prediction error signals, which guide decision-making and learning *via* the dorsal striatum (DS). Lastly, the anterior cingulate cortex (ACC) is monitoring actions and outcomes, and signals the need to engage attentional control in order to optimize behavioral output. Here, we expand upon this framework, and review our recent work in which within-task manipulations of both appetitive and aversive stimuli allow us to uncover the neural processes that contribute to the detection of outcomes delivered to a conspecific and behaviors in social contexts. Specifically, we discuss the involvement of single-unit firing in the ACC and DA signals in the NAc during the processing of appetitive and aversive events in both social and non-social contexts.

## Introduction

The neural activity of many brain regions is modulated by expected outcomes; in some cases, it is assumed that this activity corresponds to internal value representations. For example, increased neural firing in response to cues associated with reward delivery might be interpreted as reflecting the value of the anticipated reward. While this might be true, this signal might reflect its salience, which induces changes in attention, arousal, or motivation that accompany the anticipation of valued outcomes. Likewise, if an animal is not expecting reward but one is delivered, increased firing to reward delivery might be interpreted as a representation of a positive prediction error (i.e., an event that is better than predicted). However, once again, it would also be a reasonable interpretation that these changes in firing might better reflect changes in attention, arousal or motivation that accompany salient events. Manipulating both appetitive and aversive stimuli within the same task circumvents this issue, making it possible to disambiguate the encoding of genuine value predictions and prediction errors from the processing of salient events generally. If neural activity increases to cues that predict reward decrease to cues that predict an aversive event, then activity may genuinely represent value. Likewise, if activity increases and decreases to unexpected reward and punishment, respectively, then activity likely reflects signed prediction errors. Alternatively, if neural firing increases for both appetitive and aversive events, then firing might better reflect factors that co-vary with value. That is, activity might be better explained as neural correlates of cognitive functions that accompany salient events – such as heightened attention, arousal and motivation.

**FIGURE 1:**
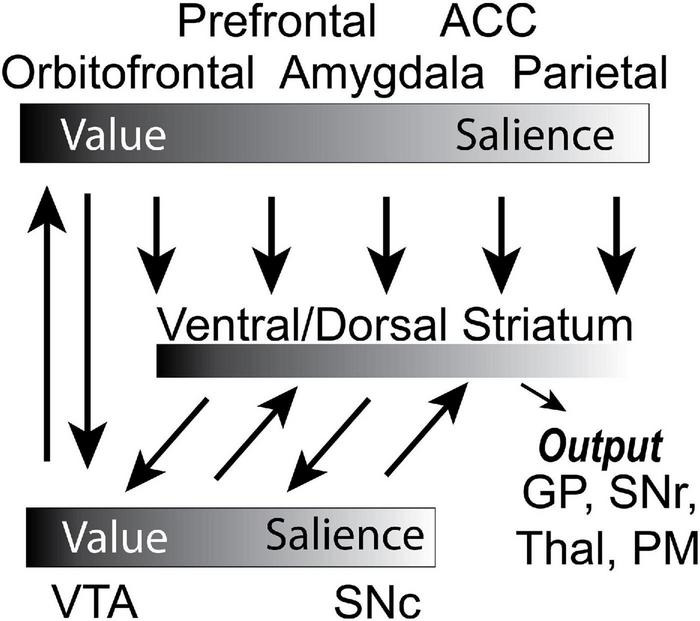
Circuit diagram of decision-making circuit. Gradients denote whether the corresponding brain region better encodes value or salience. Arrows represent the flow of information. PM, premotor cortex; SNc, substantia nigra compacta; GP, globus pallidus; Thal, thalamus; SNr, substantia nigra reticulata; VTA, ventral tegmental area. Adapted from Gentry et al. (2019).

In our previous review, we laid these ideas out in more detail, and extensively covered a number of studies that incorporated both aversive and appetitive outcomes within a paradigm in order to disambiguate value and salience signals arising from several brain areas. Note, generally, we use the term salience because salient stimuli and outcomes induce changes in attention, arousal and motivation, processes that we do not try to disambiguate here in this review. With that said, we think of attention as being more involved in the increased processing of both appetitive and aversive sensory events whereas motivation is more involved in engaging motor systems in the pursuit and avoidance of appetitive and aversive events, respectively. We described a circuit (Figure 1; Bissonette et al., 2014) in which OFC value signals are influenced by BLA encoding of the valence, intensity, and salience of appetitive and aversive events (Wassum and Izquierdo, 2015; Fiuzat et al., 2017; Brockett et al., 2021). These two areas update each other during learning, and when behavior needs to be flexible (Brockett et al., 2021). Both areas project to the NAc and VTA (Wassum and Izquierdo, 2015; Brockett et al., 2021). Separate populations of neurons in the NAc encode the value and motivational level associated with stimuli (Day and Carelli, 2007; Day et al., 2010; Bissonette et al., 2013, 2014; Wassum et al., 2013). DA neurons in the ventral tegmental area (VTA) and substantia nigra pars compacta (SNc) return prediction errors and salience signals to the NAc, and to the dorsomedial (DMS) and dorsolateral striatum (DLS) to inform goal-directed and habitual behaviors *via* spiraling connectivity (Zahm and Brog, 1992; Haber et al., 2000; Stalnaker et al., 2012; Bissonette et al., 2014; Burton et al., 2015, 2018; Keiflin and Janak, 2015; Pignatelli and Bonci, 2018). Lastly, the anterior cingulate cortex (ACC) is monitoring actions and outcomes, and signals the need to engage attentional control in order to optimize behavioral output (Weissman et al., 2005; Quilodran et al., 2008; Bryden et al., 2011; Hayden et al., 2011; Hyman et al., 2013; Wu et al., 2017; Vázquez et al., 2020).

After writing this review, it was clear that more work was needed to understand how neural systems signal appetitive and aversive stimuli, and to continue to elucidate valence-modulated signals and how they relate to subsequent behaviors or changes in cognitive states. What also became clear is that we needed to explore how the encoding of appetitive and aversive events might be modulated in social contexts. Several recent human neuroimaging studies and physiological work in animals have revealed that many of the regions that have been implicated in value processing or attention appear to also be involved in social cognition – such as in the ability to recognize emotions in others. From these studies, several areas (e.g., striatum, BLA, OFC, ACC, VTA) have emerged as being important contributors to the adaptation of behavior based on the appraisal of what is happening to others and how these outcomes extend to oneself (Blair et al., 1999; Bush et al., 2000, 2002; Kerns et al., 2004; Etkin et al., 2006; Olsson and Phelps, 2007; de Greck et al., 2008; Northoff and Hayes, 2011; Sheth et al., 2012; Báez-Mendoza and Schultz, 2013; Rudebeck and Murray, 2014; Lockwood, 2016; Allsop et al., 2018; Carrillo et al., 2019; Cox and Witten, 2019; Kim et al., 2019; Lockwood et al., 2020; Yankouskaya et al., 2020; Gangopadhyay et al., 2021).

From this body of work, it is clear that these brain areas contribute to social cognition, but–because studies exploring social decision-making focus on either appetitive or aversive outcomes, not both – there is little disambiguation of value encoding from other psychological constructs such as arousal, attention, and motivation to generally salient events. That is, neural signals related to reward evaluation may signal the valence of outcomes delivered to oneself and another, while signals related to arousal might play a role in driving attention and motivation indiscriminately toward salient social and non-social cues in the environment. Here, we provide updates with regards to appetitive and aversive encoding in non-social contexts, and how it extends to our recent work dissociating attentional signaling (i.e., unsigned signals in response to salient stimuli) from value encoding (i.e., signaling if something is good or bad) or prediction error encoding (i.e., signaling events that are better or worse than expected) in social contexts. We achieved this by manipulating both appetitive and aversive stimuli within the same paradigm, thus taking advantage of the fact that both positive (e.g., reward) and negative (e.g., shock) outcomes are arousing and attention-grabbing in both non-social and social domains. Here, we will briefly summarize key aspects of our previous review, but focus more on our recent work examining how single-unit firing in the ACC and DA signals in the NAc contribute to the processing of attention and subjective prediction errors in both social and non-social contexts.

Importantly, the circuit we will discuss in this text is not panoptic. In this review, we have focused on regions that have been studied in the context of within-task exposure to both aversive and appetitive events, allowing us to disambiguate the neural processing of value versus salience under both social and non-social contexts.

## Anterior cingulate cortex’s contribution to reward, cognitive control, and social attention – integrative processing of appetitive and aversive stimuli

The ACC has been implicated across a plethora of cognitive functions – including reward processing, conflict monitoring, arousal, surprise, feedback processing and error detection, perceptual decision-making, and attentional control (Niki and Watanabe, 1979; Carter et al., 1998; Botvinick et al., 1999, 2004; Dayan et al., 2000; Paus, 2001; Ito et al., 2003; Kerns et al., 2004; Roelofs et al., 2006; Seidman et al., 2006; Weissman et al., 2006; Seo and Lee, 2007; Croxson et al., 2009; Kennerley et al., 2009; Kennerley and Wallis, 2009; Hillman and Bilkey, 2010; Koob and Volkow, 2010; Bledsoe et al., 2011; Bryden et al., 2011; Hayden et al., 2011; Narayanan et al., 2013; Laubach et al., 2015; Kolling et al., 2016; Soltani and Izquierdo, 2019; Stolyarova et al., 2019; Brockett et al., 2020; Schneider et al., 2020; Vázquez et al., 2020; Cai and Padoa-Schioppa, 2021). However, many of these studies do not parse how these signals might be impacted by outcomes of opposite valence, and thus did not allow for the complete disambiguation of subjective value signaling from salience or attention encoding.

Generally speaking, across decision-making circuits, neural activity (e.g., in regions such as the striatum or OFC) is flexibly modulated by expected outcomes and their valence in the service of optimally driving adaptable behavior. Other cognitive processes – such as attention and motivation toward salient events – also play an important role in modulating responses toward differently valued outcomes (Bissonette et al., 2014). Attentional bias is modulated by reward outcome, and directed toward reliably predictive stimuli. Stimuli of either appetitive or aversive valence can drive attention in a way that subsequently facilitates decision-making and learning. We have found that attention-related signals in ACC can be driven by unsigned prediction errors when there are unexpected changes in outcome valence or cues that signal the need to change behavior (Bryden et al., 2011, 2019; Brockett et al., 2020; Brockett and Roesch, 2021).

We have observed neural correlates in the ACC relating to attention-based learning using a variation of a reward-guided decision-making task in which reward contingencies unexpectedly change, and reward size and delay are independently manipulated. Importantly, optimal task performance requires rats to detect unexpected changes in reward value and update behavior accordingly to select the more favorable reward outcome on free-choice trials, while maintaining accurate responding on forced-choice trials (Bryden et al., 2011; Vázquez et al., 2020). This reward-based task relies on dynamic, flexible behavior – as reward contingencies change throughout the task; successful performance requires rats to suppress prepotent responses toward previously learned associations and update their behavioral strategies.

Using this task, we previously found that ACC activity correlates with changes in attention proposed by the Pearce and Hall model of associative learning, wherein the attention given to a cue is a product of the average unsigned prediction error generated over past trials (Pearce and Hall, 1980; Bryden et al., 2011; Roesch et al., 2012). Unsigned prediction errors reflect the degree to which an outcome is unexpected, and result from the difference between the value of expected reward, versus the actual outcome. Following the model, in order for learning to occur, unsigned prediction errors should subsequently lead to increases in attention toward the cue (Pearce and Hall, 1980; Pearce et al., 1982). We have shown that ACC engagement during learning is consistent with this model (Bryden et al., 2011; Vázquez et al., 2020). ACC activity is higher after both unsigned up-shifts and down-shifts in reward value – when outcomes are better or worse than expected, respectively (Bryden et al., 2011; Vázquez et al., 2020) – a finding supported by models suggesting that the ACC serves to process valence-independent salience (Alexander and Brown, 2011; Hayes and Northoff, 2012; Yee et al., 2022).

More recent work suggests that ACC also contributes to evaluation and cognition in social contexts. Lesion and inactivation studies have implicated the ACC in vicarious fear conditioning – wherein aversion is learned through observation, instead of through direct exposure to the aversive stimulus (Jones et al., 2010; Allsop et al., 2018; Burgos-Robles et al., 2019; Carrillo et al., 2019) – and in fear learning that requires heightened attention (Han et al., 2003; Bissière et al., 2008). However, many of these studies only use stimuli of a negative valence, and thus it is difficult to discern whether ACC activity reflects global social attention or processing of prediction errors/value, or is providing outcome-specific information related to primary outcomes delivered to the self or another.

Much like non-social cues (e.g., conditioned stimuli like lights or tones), social cues can provide a wealth of information about one’s environment, and thus are often beneficial to attend to. The attentional functions that ACC is thought to contribute to in non-social contexts may also contribute to navigating behaviors in social contexts. It is known that the ACC is engaged during a number of different emotion- and social-related tasks across species. Human studies have found ACC engagement during affect-based Stroop tasks, and vicarious fear learning (Etkin et al., 2006; Olsson and Phelps, 2007). Importantly, the rodent ACC shares high degrees of functional homology with the human ACC, and connectivity studies also support this finding (Brown and Bowman, 2002; Heilbronner et al., 2016; Brockett et al., 2020).

Likewise, in monkeys, ACC contributes to cognitive functions in both non-social (Bonelli and Cummings, 2007; Wallis and Kennerley, 2010; Hayden et al., 2011; Kennerley and Walton, 2011), and social contexts (Etkin et al., 2011; Chang et al., 2013; Lockwood, 2016; Lindstrom et al., 2018; Gangopadhyay et al., 2021). Specifically, it has been shown that ACC neurons encode reward outcome information about the self, the other, or both in social contexts (Chang et al., 2013; Lindstrom et al., 2018; Noritake et al., 2018). In rodents, the ACC is not only involved in observational fear learning – responding to self-directed and socially derived cues during the task – but ACC neurons are also necessary for acquisition of the learned behavior (Jones et al., 2010; Kim et al., 2012; Allsop et al., 2018; Carrillo et al., 2019). Further, optogenetic inactivation of BLA-projecting ACC neurons results in impaired acquisition of observational fear conditioning. Clearly, the ACC contributes to social cognition across species, but the extent to what ACC signals in social contexts remains unclear.

Although primate studies focus on tasks that manipulate reward, most studies investigating the role that rodent ACC plays in social cognition have focused heavily on aversive stimuli. This focus makes sense given the role of the ACC in the affective sensation of pain – as part of the medial pain system alongside the anterior insula (Shackman et al., 2011; Xiao and Zhang, 2018; Zhao et al., 2018). Based on this connectivity – and sensitivity to both personal and vicarious pain stimuli (Singer et al., 2004) – researchers have suggested that, across species, the ACC may integrate pain and social stimuli through “emotional mirror neurons” (Preston and de Waal, 2002; Baird et al., 2011). The notion of emotional mirror neurons has been supported in rats showing subpopulations of ACC neurons that respond both to witnessing and experiencing pain, suggesting that one function of the ACC is to signal the affect of pain and fear to both the self and others (Carrillo et al., 2019). However, some of these neurons might also contribute to attention, an established non-social function of the ACC. That is – given the non-social functions of the ACC related to cognitive control, arousal and attention – it might steer attention to salient events (i.e., conspecific being shocked) regardless of their valence in social contexts as well.

To dissociate between the aforementioned signals related to value and attention, we recorded from the rat ACC in a task where presentation of stimuli predicted the valence of the outcome that was to be delivered at the end of each trial – reward, shock or nothing (Schneider et al., 2020). By manipulating both reward and shock, we determined whether activity reflected attention (both reward and shock are attention-grabbing, thus firing should be similar for both trial-types) or outcome identity (reward and shock would be differentially encoded). We showed that ACC contributes to both of these functions through different populations of neurons. However, at the population level, there was a significant positive correlation between reward- and shock-related firing – meaning that units that had increased firing to reward, also increased firing to shock (i.e., valence-independent). These findings suggest that one of ACC’s main functions in our paradigm is to heighten attention in both social and non-social contexts.

**FIGURE 2:**
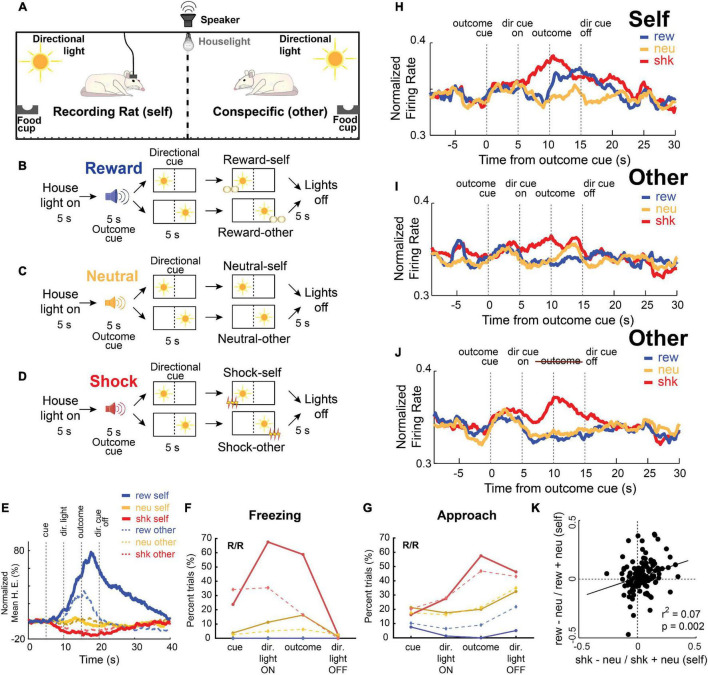
**(A–D)** Pavlovian social outcome task that interspersed reward **(B)**, neutral **(C)**, and shock **(D)** trials. Pairs of rats (recording rat and conspecific) were placed in a chamber, separated by a divider that allowed them to hear, smell, and see each other. Each trial begins with the onset of a house light; after 5 s, an auditory cue indicates the type of outcome that will be delivered (shock, reward, or none), and a 10 s directional light cue indicates which side the outcome will be delivered to (recording rat or conspecific). **(E)** Average beam breaks (food cup entry) as a percentage of trial time for each type of outcome (blue: reward; yellow: neutral; red: shock) for outcomes delivered to self (solid lines) and the conspecific (dashed lines). **(F)** Percentage of trials recording rats froze during each epoch for each type of outcome (blue: reward; yellow: neutral; red: shock) for outcomes delivered to self (solid lines) and the conspecific (dashed lines). **(G)** Same as panel **(F)**, but for rat approach to the divider – defined as when the recording rat moved toward, or was actively interacting at, the divider. **(H–J)** Normalized average firing rate of all recorded ACC neurons (*n* = 139) across each trial type (blue: reward, red: shock, yellow: neutral) for outcomes delivered to self **(H)** and the conspecific **(I,J)**. In panel **(J)**, predictive cues were used but no outcomes were delivered to the conspecific. **(K)** Correlation of ACC activity on shock-self and reward-self trials. Adapted from Schneider et al. (2020).

The specifics of the task are illustrated in Figure 2. In this study, rats were placed in opposite sides of a modified shuttle box, which was separated by a mesh divider so they could still observe, approach, and sense their conspecific. The walls opposite to the divider were equipped with a directional cue light, a food cup, and a shock grid (Figure 2A). Five seconds after onset of the illumination of a house light, an auditory stimulus (5 s) predicted delivery of one of three possible corresponding outcomes (either a sucrose pellet, foot-shock, or nothing randomly interleaved), and a cue light predicted whether that outcome would be delivered to either the recording rat (self) or the conspecific (other). After presentation of the directional light for 5 s, the outcome (reward, shock or nothing) was delivered to the same side as the illuminated light cue (Figures 2B–D). When reporting the results below, we will refer to “Self” trials as trials during which the outcome was delivered to the recording rat, whereas “Other” trials refer to trials in which the outcome was delivered to the conspecific.

In this paradigm, rats increased and decreased beam-breaks in to the food cup on reward-self (blue) and shock-self (red) trials relative to neutral (yellow) trials (Figure 2E). Rats also froze more to cues that predicted shock-self and shock-other (Figure 2F), and then would approach each other toward the end of shock trials relative to neutral trials (Figure 2G). Critically, in this paradigm, reward and shock trials have opposite valences, but both outcomes are arousing and attention grabbing, thus allowing us to dissociate value encoding from attention in ACC.

Figures 2H–J show the average activity over all recorded ACC neurons (*n* = 139) across each trial-type. As in previous studies, we saw increases in firing during shock trials compared to neutral (yellow) in trial blocks for both shock-self and shock-other. Notably, these firing increases on shock-other trials were not observed during sessions in which the conspecific was not present, suggesting that the presence of the conspecific was necessary for the observed increases in activity. Further, firing was also present on shock-other trials, even when the shock was omitted (Figure 2J). Importantly, firing across the population was not only higher for shock compared to neutral trials, but was also higher during reward-self trials. Further, the two were positively correlated – cells that exhibited higher firing during shock trials also exhibited higher firing during reward trials (Figure 2K). Thus, these findings suggest that the ACC, under this paradigm, contributes to attention-related processing of social and non-social cues. Although activity at the population level in ACC was elevated for both reward and shock – suggesting that overall function of ACC is closely aligned with attention in this paradigm – other neurons did respond selectively to shock or reward delivered to the Self or the Other (Schneider et al., 2020).

Thus, consistent with previous reports, we found that ACC firing was modulated by aversive stimuli delivered to both self and other. Additionally, we found that – while the activity of some of these neurons genuinely reflected outcome identity (i.e., reward or shock) – the population as a whole responded similarly for both reward and shock, as well as for cues that predicted their occurrence. Similar to the role it plays in non-social decision-making, we conclude that ACC processes information about outcomes (i.e., identity, recipient) in the service of promoting attention in social contexts.

## Basolateral amygdala – multidimensional encoding of negative and positive valence

Due to the BLA’s bidirectional connectivity with the ACC, and its involvement in learning and social processing, here we briefly review the BLA’s contribution to these processes. At the level of single neurons, BLA activity is modulated by the value and predictability of outcomes (Roesch et al., 2010). These signals are similar to the aforementioned attentional signals we found in the ACC; however, an important distinction is that ACC activity increased prior to trial events following unexpected switches in reward contingencies (Bryden et al., 2011), while BLA signaled unsigned prediction errors at the time of reward (Roesch et al., 2010). The bidirectional nature of ACC and BLA connectivity suggests that this circuit is responsible for the detection of prediction errors, and the subsequent attentional increases that are necessary for dynamic learning to occur (Bryden et al., 2011). Supporting this idea, other studies have shown that disruption of this connectivity results in impaired decision-making and behavioral flexibility (Murray, 2007; Salzman and Fusi, 2010; Yang et al., 2016; Yizhar and Klavir, 2018; van Holstein et al., 2020; Brockett et al., 2021). Further, during aversive conditioning, unsigned prediction error signals in the primate BLA are transmitted to the ACC *via* synchronous theta phase coupling (Taub et al., 2018). These results are consistent with a study that was able to find that these theta oscillations were positively correlated with the rate of fear learning in humans (Chen et al., 2021). Together, these studies suggest that heightened activity in the amygdala may be helping synchronize ACC activity in a way that transfers error signal information, subsequently leading to the increases in attention that facilitate flexible learning (Taub et al., 2018; Chen et al., 2021).

However, many single-unit recording studies have found evidence that BLA neurons also signal valence, independent from attention (Salzman and Fusi, 2010; Gore et al., 2015; O’Neill et al., 2018). For example, during performance of a Go/No-Go task, rats were trained to associate one odor (“go”) with responding into a fluid well to receive a reward. Another odor (“no-go”) was associated with an aversive outcome (quinine delivery instead of sucrose) (Schoenbaum et al., 1998). Thus, rats learned to withhold prepotent responding on “no-go” trials. Researchers found that 36% of recorded BLA neurons differentially encoded outcome identity – by developing selectivity toward a cue associated with a particular valence (Schoenbaum et al., 1998).

While the intention of each of these accounts of BLA function (e.g., fear, reward, valence, salience, prediction errors) have led to insights about the BLA’s role in behavior, these unidimensional explanations may oversimplify function, constrained by paradigms designed to tightly control and monitor all aspects of behavior so as to better correlate neural signals with learning and behavior. Recordings from amygdala have often revealed highly complex selectivity (Nishijo et al., 1988; Belova et al., 2007; Rigotti et al., 2013; Fusi et al., 2016; Kyriazi et al., 2018, 2020; Putnam and Gothard, 2019; Gothard, 2020) that is reminiscent of selectivity found in frontal brain regions. For example, Kyriazi and colleagues have mapped conditioned stimuli (CS)- and conditioned response (CR)-related activity toward appetitive and aversive stimuli in BLA neurons to determine whether individual cells in the amygdala encode CS, CR, or both. During performance of a Risk-Reward Interaction (RRI) task – which required rats to respond to both reward predicting and shock predicting cues – researchers found that single BLA neurons concurrently and independently encode CSs (signaling both appetitive and aversive outcomes) and learned CRs (both approach and avoidance behaviors), suggesting that most BLA neurons heterogeneously encode multiple task and stimulus features (Kyriazi et al., 2018). Other studies in primates learning to associate reward or punishment with two different behavioral contexts showed that activity in amygdala reflected context representations – in addition to encoding stimulus identity and reinforcement expectations (Saez et al., 2015).

Along with its well-studied, critical component in non-social learning and decision-making, the amygdala has gained considerable attention for its contributions to social cognition. For example, as mentioned above, during observational fear conditioning, inhibition of BLA-projecting ACC neurons prevented vicarious learning during the task (Allsop et al., 2018). BLA recordings obtained during the procedure suggested that ACC inputs were modulating the baseline activity of BLA neurons, potentially facilitating association of perceived social cues during the task. Consistent with those findings, it has been shown that inactivation of BLA alters the investigation of social cues in female rats, suggesting that it is required for the prioritizing of social cues (Song et al., 2021). Notably, it is not just basolateral portions of the amygdala that have been linked to social cognition; other studies have shown that the central and medial amygdala subregions contribute to various social behaviors (Hong et al., 2014; Minami et al., 2019; Andraka et al., 2021; Hu et al., 2021). Unlike these regions, the BLA is thought to be upstream from more central amygdala structures, activating them differentially to achieve different behavioral outcomes (Janak and Tye, 2015). Together, previous non-social and social findings in BLA have spotlighted it as a potential area for the processing of social outcome valence, especially because it is thought to encode the affective perception of pain at the level of neural ensembles (Corder et al., 2019; Brockett et al., 2021).

## Dopaminergic involvement – prediction errors and salience processing

Lesion studies have shown that the aforementioned signals in the BLA appear to be partially dependent on midbrain dopamine – specifically from the VTA – which, unlike the signals we described for BLA, are thought to encode signed reward prediction errors (Esber et al., 2012). Signed reward prediction error signals are generated when there are differences between expected and actual outcomes – facilitating the updating of response-outcome associations so that learning can occur (Schultz et al., 1997; Keiflin and Janak, 2015; Nasser et al., 2017). While a plethora of studies have documented the way in which dopaminergic signals are modulated by the value of stimuli and transmit prediction error related information (Schultz, 1998, 2010; Collins et al., 2016; Eshel et al., 2016; Berke, 2018; Walton and Bouret, 2019), the DA signal does not always differentiate between appetitive or aversive stimuli and outcomes, reflecting their salience (Kutlu et al., 2021).

We have addressed these issues recently by looking at dopamine release in the NAc in the context of unavoidable and avoidable shock. While DA function has been widely implicated in function pertaining to reward, a growing literature has examined how DA contributes to aversive associative learning. Several studies have shown a valence-dependent DA response profile wherein aversive stimuli decrease DA firing and release, while the omission of expected aversive outcomes result in an increased DA response (Roitman et al., 2008; Darvas et al., 2011; Badrinarayan et al., 2012; Budygin et al., 2012; Oleson et al., 2012; Oleson and Cheer, 2013; Volman et al., 2013). With that said, increased DA activity has also been shown to occur in direct response to aversive physical stimuli – including tail pinches, foot shocks, or air puffs (Abercrombie et al., 1989; Young et al., 1993; Wilkinson et al., 1998; Brischoux et al., 2009; Matsumoto and Hikosaka, 2009; Budygin et al., 2012) and activation of both striatal D1 and D2 receptors is necessary for the formation of fear memories (Fadok et al., 2009; Ikegami et al., 2018).

It has been suggested that some of the heterogeneity seen in the response profiles of midbrain DA neurons to aversive stimuli is actually due to the incorrect classification of DA vs. non-DA neurons (Ungless et al., 2004; Ungless and Grace, 2012). Other studies have shown that while DA neurons in the dorsal VTA were inhibited by aversive foot shock, DA neurons in the ventral portion of VTA were excited by the same foot shock (Brischoux et al., 2009). Further heterogeneity has been described in primates along the dorsoventral and mediolateral axes (Bromberg-Martin et al., 2010), with more medial and ventral midbrain DA neurons signaling reward prediction errors, and more dorsolateral SNc neurons encoding salience signals (Matsumoto and Hikosaka, 2009; Bromberg-Martin et al., 2010).

Heterogeneity of DA signals has also been described in mice (Cohen et al., 2012; Lammel et al., 2014). In these studies, DA neurons were identified optogenetically, and were shown to respond to reward predictive cues, rewards, and reward omissions conforming to reward prediction errors. Other reports from the same group demonstrated that the firing of many DA neurons to air-puffs and cues that predict them were biphasic, suggesting that some of the reported excitatory DA neuron responses to aversive stimuli may be due to an initial excitatory response, followed by longer lasting decreases in activity (Tian and Uchida, 2015). Further, DA neurons more reliably signal prediction errors when aversive stimuli are presented in different reward contexts within the same task (Tian and Uchida, 2015). VTA projections are also an important consideration. Optogenetic and addiction studies by Lammel and colleagues have shown that VTA efferents projecting to NAc are involved in appetitive processing, while efferents to the prefrontal cortex are implicated in aversion (Lex and Hauber, 2008; Lammel et al., 2011, 2012; Ben-Ami Bartal et al., 2014).

It has also been proposed that DA neurons take prediction errors as an input, transform the information, and signal salience as an output (Berridge, 2007) and that signals differ as a result of variable DA innervation, regional differences in dopamine sensitivity, and variations in signal kinetics across striatal regions (Wickens et al., 2007; Saddoris et al., 2015). Consistent with these ideas, recent observations demonstrate that changes in NAc DA release can be dissociated from changes in DA neuron action potential firing (Hamid et al., 2016; Berke, 2018; Mohebi et al., 2019), and it has been hypothesized that DA encoded value signals can underlie both prediction errors and salience (Berke, 2018). Further support for a role of presynaptic regulation in sculpting DA signals comes from fiber photometry studies examining neuron terminals in various regions of the striatum, showing that signals match prediction error signals and salience signals in the ventral and dorsal striatum, respectively, for both appetitive and aversive related stimuli (Menegas et al., 2017; Yuan et al., 2019). Interestingly – in both the dorsomedial and dorsolateral striatum – prediction error (PE) signals were more prominent for reward-related stimuli, whereas salience signals were more evident for punishment-related stimuli (Yuan et al., 2019).

While many of these studies have focused on unavoidable shock or punishment, others have examined the DA system in the context of avoidance. In a typical avoidance paradigm, an animal is presented with a warning cue (e.g., tone) which precedes an aversive outcome (e.g., shock) unless a required behavioral response (e.g., lever press) is performed. This type of behavior has long been thought to rely on both Pavlovian (i.e., fear of warning cue) and instrumental learning (i.e., terminating the warning cue reinforces the behavior). A number of studies have shown that DA is important for avoidance. For example, lesioning midbrain DA neurons or DA terminals in NAc specifically prevents animals from acquiring avoidance, while leaving other escape behaviors intact (Cooper et al., 1974; Fibiger and Phillips, 1974; Zis et al., 1974; Amalric and Koob, 1987; McCullough et al., 1993). Further, tonic DA levels tend to be higher in the striatum during both the acquisition and maintenance of avoidance behavior (McCullough et al., 1993; Dombrowski et al., 2013), broadly inhibiting DA using antagonists prevents animals from acquiring avoidance (Fibiger et al., 1975; Beninger et al., 1980; Arnt, 1982; Wadenberg et al., 1990; Inoue et al., 2000; Fadok et al., 2009), and D1-knockout mice show impaired acquisition of fear conditioning and extinction (Ikegami et al., 2018).

These studies all suggest that DA release increases during avoidance. However, in computational models of the two-factor theory of avoidance (Dayan, 2012; Lloyd and Dayan, 2019), positive prediction errors are generated when aversive events are successfully avoided, because the value of the outcome (no shock) is referenced to the estimated value (shock). Consistent with this model, it has been shown that DA release increases to the warning cue only when animals subsequently avoided shock, and that no increases in DA release were present to cues that were followed by failed presses, or by lever presses that terminated shock after it had already begun (escape response). Instead, during escape responses there was a significant decrease in DA between the cue presentation and lever press (Oleson and Cheer, 2013). It is possible, then, that increases in phasic DA released to shock avoidance cues and to the receipt of safety could be analogous to cues predicting reward and reward delivery – wherein successful avoidance of an expected aversive consequence is rewarding. The researchers also went on to show that predicted unavoidable shock paused DA release. Combined, these results suggest that increased cue-evoked DA release in the NAc predicts successful shock avoidance, whereas a pause in DA transients occurs during the presentation of unavoidable aversive stimuli (Roitman et al., 2008; Darvas et al., 2011; Badrinarayan et al., 2012; Oleson and Cheer, 2013; Volman et al., 2013).

Although there is now a considerable body of literature that has studied DA contribution to reward approach and active shock avoidance, very few have set out to do so explicitly within the same task. To address this issue, we developed at task in which rats experienced three separate cues predicting the possibility for reward, the possibility for shock, or no change in the environment. After presentation of the cue, a lever was extended into the chamber, at which point rats could press to either receive a sucrose pellet (positive reinforcement) or to prevent foot shock (negative reinforcement) depending on the identity of the predictive cue. If the rat failed to press the lever, no food reward was delivered or shock was commenced (Gentry et al., 2016, 2019).

We found that DA release within the NAc increased to both reward approach and shock avoidance cues. Further, we found significant positive correlations between DA release on reward and shock trials relative to neutral trials during the presentation of cues and during the lever press. This work confirmed that, at least within the NAc core, local DA release can signal the need for approach or avoidance behavior, and tracks the value of each cue. This result coincides with prior results from Oleson et al. showing that DA release in NAc core predicts successful avoidance, and is inhibited by unavoidable shock (Oleson et al., 2012). We have recently replicated these results in similar tasks that not only manipulated reward and shock within the same paradigm, but also manipulated reward and shock that would be delivered to a conspecific nearby as we will describe below.

In these studies, we have shown that DA release in NAc reflects the subjective value placed on appetitive and aversive events as opposed to the objective value of the event itself. To achieve this, we recorded DA release in the NAc using fast-scan cyclic voltammetry (FSCV) in two different social paradigms – one that used Pavlovian cues to predict reward or unavoidable shock to either the recording rat or to a nearby conspecific, and a second that was instrumental – allowing the recording rat to refrain from the pursuit of reward in order to avoid harmful consequences (i.e., footshock) for itself or the conspecific. In both studies, we found signs that rats were “empathetic” and/or “prosocial” as previously described; however, we also showed that rats are often not overly empathetic or prosocial, in that their behavior and DA signals were modulated far more by potential reward and shock that occurred directly to them as opposed to their conspecific, typically reflecting a prioritization of their own physical state over their conspecific’s across multiple within- and across-task comparisons (Kashtelyan et al., 2014; Lichtenberg et al., 2018; Schneider et al., 2020).

**FIGURE 3:**
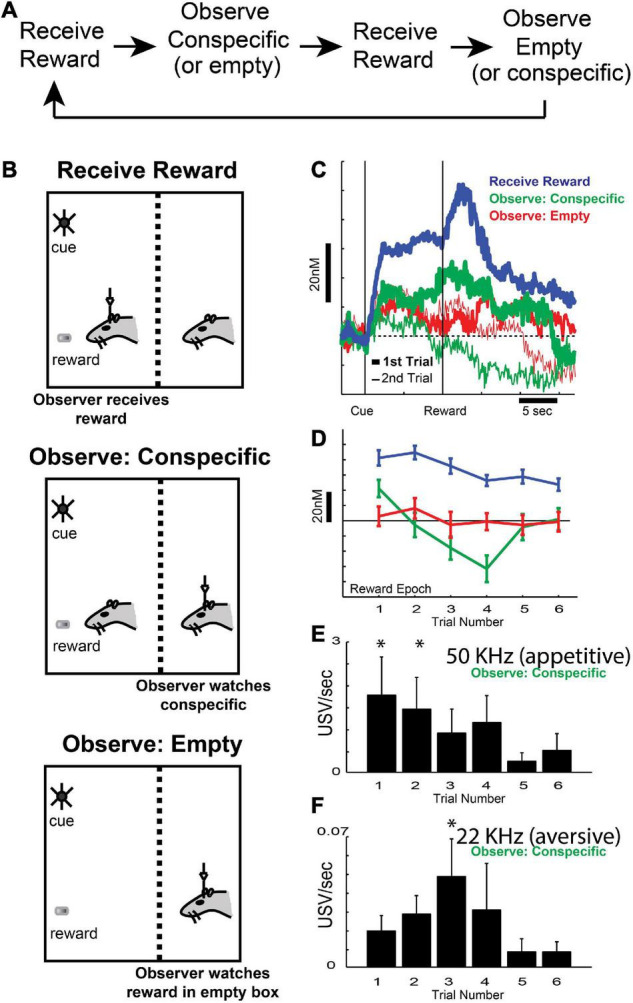
**(A,B)** Task design, with an example of the order of blocks within a session. Reward (sucrose pellet) was delivered 10 s following the onset of cue lights. Each session consisted of three block types **(B)**: (blue: recording rat receives reward, green: recording rat observes conspecific receiving reward, or red: recording rat observes a sucrose pellet being delivered to an empty chamber), with an average of 15 trials per block. Rats were able to observe, smell, and hear the conspecific through a wire mesh. **(C)** Average dopamine concentration in NAc over time during the first (thick lines) and second (thin lines) trial of each trial type (blue: recording rat receives reward, green: recording rat observes conspecific receiving reward, or red: recording rat observes a sucrose pellet being delivered to an empty chamber). **(D)** Average dopamine release for all trial types (blue: recording rat receives reward, green: recording rat observes conspecific receiving reward, or red: recording rat observes a sucrose pellet being delivered to an empty chamber) during the reward epoch of the first six trials within the block. **(E,F)** 50 **(E)** and 22 kHz **(F)** ultrasonic vocalization rates for the first six trials during the reward epoch (2 s following reward delivery to the conspecific). Asterisks denote significant difference between indicated trial and the last trial; Wilcoxons, *p* < 0.05. Adapted from Kashtelyan et al. (2014).

Our first contribution to this field was to show that DA release is modulated both by delivery of reward to the self and a conspecific, and by a mixture of affective states during the observation of conspecific reward (Figures 3A–D) – initially exhibiting increases in appetitive calls (50 kHz; Figure 3E) at the beginning of reward-other blocks, then exhibiting increases in aversive calls (22 kHz; Figure 3F) as reward-other trials continued (Kashtelyan et al., 2014). Thus, when rats first observed another rat receive reward, it found it to be appetitive, but after experiencing that event several times, the rats observing the other rat receive reward found it to be aversive, perhaps reflecting the understanding that it would not be the one receiving the reward. Like ultrasonic vocalizations (USVs; Figures 3E,F), DA signals (Figures 3C,D) were modulated by delivery of reward to the conspecific, which mapped onto the emotional state associated with the conspecific receiving reward (i.e., initially appetitive, then aversive; Figures 3E,F; Kashtelyan et al., 2014). These results demonstrated that the appetitive and aversive states associated with conspecific reward delivery modulated DA signals related to learning in social situations in a signed fashion, reflecting the subjective self-interested evaluation of rewards delivered to conspecific.

**FIGURE 4:**
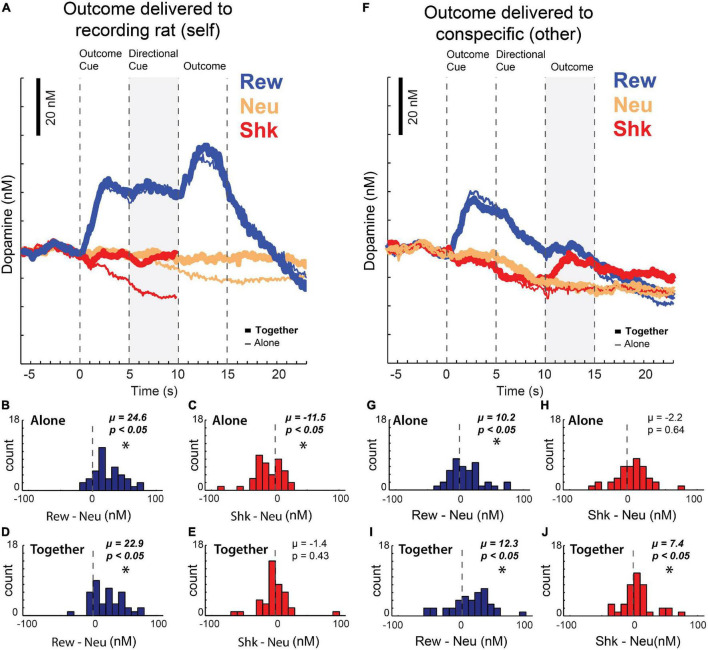
**(A)** Average dopamine concentration over time on reward (blue), neutral (yellow), and shock (red) trials when the outcome was delivered to the recording rat – when the rat was with a conspecific (thick line) or alone (thin line) during performance of the task described in Figure 2. Shock trials are truncated due to noise artifacts. **(B–E)** Reward (reward minus neutral) and shock (shock minus neutral) indices of dopamine release during the directional cue epoch (gray bar in **A**) across sessions when the recording rat was alone **(B,C)** or with a conspecific **(D,E)**. **(F)** Same as **(A)**, but when the outcomes were being delivered to the conspecific **(G–J)** same as **(B–E)**, but when the outcomes were being delivered to the conspecific instead of to the recording rat (reward epoch = gray bar in **F**). Asterisks denote significant shifts from zero; Wilcoxons, *p* < 0.05. Adapted from Lichtenberg et al. (2018).

After publishing these results, we replicated these findings and extended them using the newly designed Pavlovian task described above in the context of ACC recording (Figures 2A–D), that not only manipulated reward, but also shock. Unlike firing in ACC, which we suggested reflected attention to salient cues, average DA release over all sessions increased to cues and rewards delivered to the recording rat (Figures 4A–E; blue) but was lower during the presentation of cues that predicted unavoidable shock (Figure 4A; red; note that due to shock artifact we could not record DA release during shock-self trials during this study). Thus, as described previously, DA release increased and decreased during cues that predicted appetitive and aversive events, respectively, reflecting value and/or errors in reward prediction (Schultz et al., 1997; Roitman et al., 2008; Bromberg-Martin et al., 2010; Roesch et al., 2010; Schultz, 2010, 2015; McCutcheon et al., 2012; Oleson et al., 2012; Wenzel et al., 2018). Interestingly, we also found that the decline of DA release induced by the presentation of shock-self cues was attenuated in the conspecific’s presence (Figure 4A; thick red vs. thin red; cue epoch: gray bar) consistent with the reductions in behavioral measures of fear observed on these trials, suggesting that fear is buffered by social interaction.

These data show that DA release increases and decreases to cues that predict reward and shock to oneself, respectively, reflecting subjective value. Notably, when reward and shocks were directed at the conspecific, instead of the recording rat, DA release also tracked the subjective value the recording rat placed on those stimuli, as opposed to their physical nature. We found that there was an increase of DA release similar to what was observed during reward-self trials; however, after directional cue presentation – when rats became aware that the conspecific would receive the reward – DA declined to pre-cue levels (Figure 4F). As a result, DA release was significantly lower when reward was delivered to the conspecific compared to when reward was delivered to the recording rat, consistent with our previous work showing that rats find rewards delivered to a conspecific not appetitive with repeated exposure. Remarkably, the opposite was true on shock-other trials – prior to shock delivery, we found that DA release was similar for shock-self and shock-other trials. However, after shock was delivered to the conspecific, DA release actually increased relative to when the rat was alone (Figure 4F; red; Figures 4G–J), possibly reflecting that shock delivered to the other is better than shock delivered to oneself and/or confirming that the shock will not be directed to the recording rat *via* the observed distress of the conspecific. Together, these findings suggest that DA is modulated by the social context in which appetitive and aversive outcomes occur.

**FIGURE 5:**
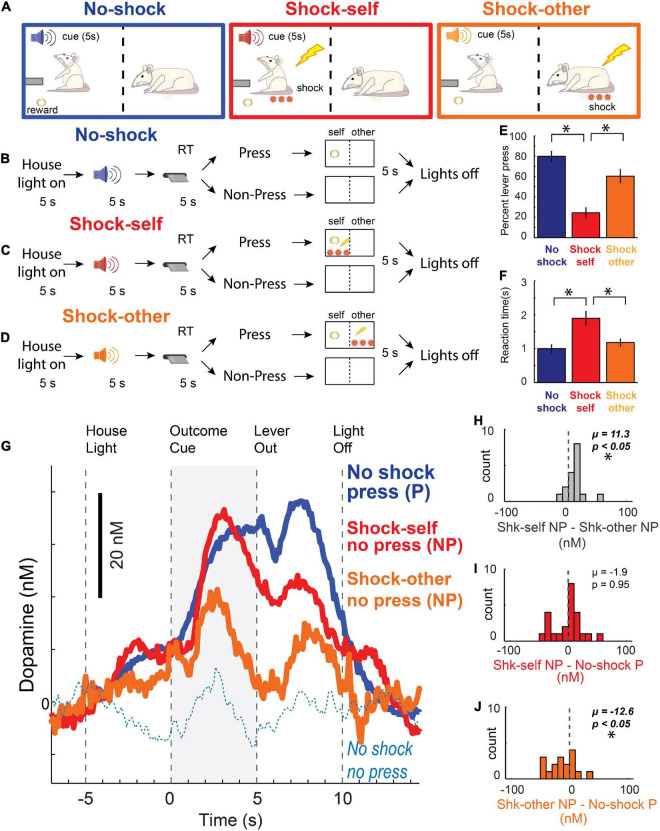
**(A–D)** Instrumental social distress task design. Recording rats were presented with a lever 5 s after presentation of 1 of 3 different auditory stimuli. Lever pressing always led to sucrose reward, but on some trials, different auditory stimulus predicted that lever-pressing would also lead to shock delivery – either to oneself (red; Shock-Self), or to the conspecific (orange; Shock-Other). After lever extension, rats had 5 s to press; otherwise, the lever retracted and no outcomes were delivered. Thus, on “Shock-Self” trials, if the recording rat pressed, it received reward and shock. On “Shock-Other” trials, if the recording rat pressed, it received reward and the conspecific was shocked. If they did not press on “Shock” trials, they avoided shock for themselves or the conspecific (depending on the trial-type), at the cost of not receiving reward. On “No-Shock” trials recording rats pressed the lever for reward with no threat of shock to oneself or the conspecific (blue). **(E)** Percentage of lever presses per trial type. **(F)** Latency to lever-press. **(E–F)** Wilcoxon, *p* < 0.05. **(G)** Dopamine release (nM) in NAc over time (s) across each trial type. Blue solid = recording rat pressed the lever for reward; Blue dashed = recording rat failed to press the lever for reward; Red = recording rat did not press during shock-self trials, thus avoided shock for oneself and forfeited reward on that trial; Orange = recording rat avoided shock to the conspecific at the cost of giving up reward for oneself. **(H–J)** Indices of DA release for each trial type during the outcome cue epoch (gray bar in **G**) across sessions: **(H)** shock self (*no press*) minus shock other (*no press*); **(I)** shock self (*no press*) minus no shock (*press*); **(J)** shock other (*no press*) minus no-shock (*press*). **(H–J)** Asterisks denote significant shifts from zero; Wilcoxons, *p* < 0.05. Adapted from Lichtenberg et al. (2018).

Taken together, the analysis of behavior and DA release during the Pavlovian Social Distress Paradigm suggested that DA release better reflected the subjective valuation of appetitive and aversive events, as opposed to the objective value of stimuli and outcomes or their salience. However, since that paradigm was completely Pavlovian, rats were never given the opportunity to perform “prosocial” acts that relieved distress for others, making it difficult to truly characterize the DA response and the nature of their behavior. To address this issue, we had a different cohort of rats perform an Instrumental Social Distress Task, illustrated in Figures 5A–D. During performance of this task, rats were presented with a lever 5 s after presentation of an auditory stimulus. Lever pressing always led to sucrose reward, but on some trials the auditory stimulus predicted that lever-pressing would also lead to a shock being delivered either to oneself or to the conspecific. After extension of the lever, rats had 5 s to press; otherwise, the lever retracted and no outcome was delivered. Overall, rats were less likely (Figure 5E) and took longer (Figure 5F) to lever press on shock-other trials relative to non-shock trials, consistent with “prosocial” behavior seen in other studies (Atsak et al., 2011; Ben-Ami Bartal et al., 2011, 2014; Burkett et al., 2016; Meyza et al., 2017).

Although cues that predict unavoidable footshock suppress DA release (as in the Pavlovian Social Outcome Paradigm) – as we have described above, cues that predict avoidable shock and avoidance of shock itself, can increase DA release to a similar degree as cues that predict reward delivery (Oleson et al., 2012; Oleson and Cheer, 2013; Gentry et al., 2016; Wenzel et al., 2018). Notably, we replicated those results in this task. Thus, unlike cues that predict unavoidable shock – which are aversive and suppress DA release – cues that predict avoidable shock increase DA release, reflecting the value of successfully avoiding an aversive outcome. Remarkably – as during non-press shock-self trials – a similar, yet reduced, pattern of DA release emerged, with DA release occurring to the cue and during the absence of the shock (Figures 5G–J). This reduced DA release when the conspecific was saved during shock-other trials, relative to when the recording rats avoided shock for oneself, likely reflects lesser concern for the conspecific compared to oneself as demonstrated by significantly less avoidance on shock-other compared to shock-self trials (Figures 5E,F).

## Orbitofrontal cortex–evaluation of expected rewards delivered to self and others

In our original review we heavily discussed the OFC – as it is a region that is critical for encoding expectations about future appetitive and aversive outcomes, which is imperative for guiding learning and flexible decision-making (Schoenbaum et al., 1998; Roesch and Olson, 2004; Schoenbaum and Roesch, 2005; Plassmann et al., 2010; Morrison et al., 2011; Morrison and Salzman, 2011; Bissonette et al., 2014). However, this brain region has yet to be examined in a social paradigm that manipulates both appetitive and aversive stimuli. Further, in non-social tasks that have presented both appetitive and aversive stimuli, it has been shown that OFC activity reflects value as opposed to motivation (Roesch and Olson, 2004; Bissonette et al., 2014). It has also been shown that in addition to populations of OFC neurons that represent value, the activity of other neurons reflects the actual offers being made or the option that will be eventually chosen during performance of a choice task (Padoa-Schioppa and Assad, 2006; Morrison et al., 2011; Bissonette et al., 2014). Collectively, these studies have shown that OFC has all the signals necessary, at the single unit level, to make reward-guided decisions, as opposed to facilitating behavior through general motivational mechanisms. Recent work demonstrates that this is also true in the social domain (Machado and Bachevalier, 2006; Azzi et al., 2012; Chang et al., 2013). Further, studies involving OFC disruption in humans (Bechara et al., 2000; Blair, 2010; Forbes and Grafman, 2010), rats (Rudebeck et al., 2007; Kuniishi et al., 2017; Jennings et al., 2019), and non-human primates (Machado and Bachevalier, 2006) report impairments in social behaviors. Lastly, OFC is also activated during mutual cooperation in a prisoner’s dilemma-type task (Decety et al., 2004) and OFC neurons signal the value of rewards that are to be delivered to the self and others, with an emphasis on self-directed reward (Chang et al., 2013). Future work examining OFC in tasks that manipulate both appetitive and aversive domains are necessary to determine how its signals are impacted by aversive stimuli directed to others in relation to reward and shock delivered to the self.

## Other regions of interest – future directions

There are a multitude of other regions also involved in the processing of rewarding and aversive events in non-social and social tasks that we do not have the space to discuss here [e.g., hypothalamus, lateral habenula, rostral tegmental nucleus, dorsal raphe, pedunculopontine tegmental and laterodorsal tegmental nucleus, hippocampus, insula, and locus coeruleus (Sara and Segal, 1991; Jhou et al., 2009; Wang et al., 2017; Noritake and Nakamura, 2019)]. Some of these regions have been studied in the context of both appetitive and aversive events within the same paradigm, but not in the context of social processing, whereas others have been studied in the social domain, but their firing has not yet been fully characterized. For example, using fiber photometry, researchers found excitatory responses in the lateral habenula to aversion-predicting cues, and inhibitory response patterns to reward-predicting cues (Wang et al., 2017). Future studies should look into whether these response patterns extend to social contexts. Another region of interest is the insula, which has been implicated in social cognition across species (Droutman et al., 2015; Rogers-Carter et al., 2018). Along with the ACC, it forms an integral part of the “salience network,” which plays an important role in selecting which stimuli an organism attends to Qadir et al. (2018) and Seeley (2019). Tasks that apply both appetitive and aversive stimuli might better uncover the nature of signals observed in this region and how it relates to attentional signals observed in ACC in both social and non-social contexts. Finally, the locus coeruleus (LC) – a region with widespread projections throughout the entire central nervous system – consists of neurons that synthesize norepinephrine and play a major role in arousal and attention (Breton-Provencher et al., 2021) is also thought to contribute to social stress (Reyes et al., 2015). Future studies should investigate how norepinephrine release in ACC might modulate attention to social cues.

## Conclusion

In our findings, both ACC firing and attention to the task increased on trials immediately following unsigned prediction errors, (i.e., trials following outcomes that were either better or worse than expected) and when rats anticipate and receive both reward and shock (Bryden et al., 2011; Hayden et al., 2011; Schneider et al., 2020; Vázquez et al., 2020). The observed alterations in attentional control likely impact decision-making in social and non-social context, particularly with regards to being able to dynamically update behavior in environments that are uncertain. These attentional signals in ACC modulate and are modulated by signed and unsigned prediction error signals from VTA and BLA, and evaluative expectancy signals in OFC. The BLA’s involvement in both appetitive and aversive processing also likely plays a pivotal role in context-dependent associative learning, and assigning salience and value to cues that signal specific appetitive and aversive outcomes (Brockett et al., 2021), whereas DA signals arising in VTA might better reflect errors in reward predictions based on the subjective value that the animal places on outcomes delivered to oneself, as well as those delivered to others. Notably, DA signals arising from different subregions of the midbrain and/or those that project to different regions in striatum and prefrontal cortex might better reflect salience, or be modified downstream depending on task context and innervation.

## Author contributions

DV and MR wrote the manuscript. KS edited and provided figures and data. All authors contributed to the article and approved the submitted version.
